# Anesthetics Rapidly Promote Synaptogenesis during a Critical Period of Brain Development

**DOI:** 10.1371/journal.pone.0007043

**Published:** 2009-09-16

**Authors:** Mathias De Roo, Paul Klauser, Adrian Briner, Irina Nikonenko, Pablo Mendez, Alexandre Dayer, Jozsef Z. Kiss, Dominique Muller, Laszlo Vutskits

**Affiliations:** 1 Department of Neuroscience, Faculty of Medicine, University of Geneva, Geneva, Switzerland; 2 Department of Anesthesiology, Pharmacology and Intensive Care, University Hospital of Geneva, Geneva, Switzerland; 3 Department of Adult Psychiatry, University Hospital of Geneva, Geneva, Switzerland; Institut de la Vision, France

## Abstract

Experience-driven activity plays an essential role in the development of brain circuitry during critical periods of early postnatal life, a process that depends upon a dynamic balance between excitatory and inhibitory signals. Since general anesthetics are powerful pharmacological modulators of neuronal activity, an important question is whether and how these drugs can affect the development of synaptic networks. To address this issue, we examined here the impact of anesthetics on synapse growth and dynamics. We show that exposure of young rodents to anesthetics that either enhance GABAergic inhibition or block NMDA receptors rapidly induce a significant increase in dendritic spine density in the somatosensory cortex and hippocampus. This effect is developmentally regulated; it is transient but lasts for several days and is also reproduced by selective antagonists of excitatory receptors. Analyses of spine dynamics in hippocampal slice cultures reveals that this effect is mediated through an increased rate of protrusions formation, a better stabilization of newly formed spines, and leads to the formation of functional synapses. Altogether, these findings point to anesthesia as an important modulator of spine dynamics in the developing brain and suggest the existence of a homeostatic process regulating spine formation as a function of neural activity. Importantly, they also raise concern about the potential impact of these drugs on human practice, when applied during critical periods of development in infants.

## Introduction

Formation, elimination and remodeling of excitatory synapses on dendritic spines are continuously active processes that shape the organization of synaptic networks during development. In vivo experiments have shown that these processes are developmentally regulated, and are under the control of experience-driven neuronal activity [1]–[5]. Accumulating experimental works demonstrate that, during critical periods of development, both environmental, genetic and pharmacological interference with physiological neuronal activity can markedly and permanently alter wiring patterns and, thereby, information processing in the central nervous system (CNS) [6]–[8]. An important parameter regulating these processes is the balance between excitation and inhibition [9]. Alteration of this balance through interference with the function of local inhibitory circuits determines the characteristics and spacing of input segregation for ocular dominance columns formation and also controls the onset of critical periods by regulating perisomatic GABA responses [10]–[12]. The level of inhibition present in developing cortical networks plays therefore an important role in fine-tuning cortical circuitry to experience [13]. In line, functional deficits in neurodevelopmental disorders, such as the Down and the Rett syndrome, or autism spectrum disorders have been proposed to be linked to a shift in the balance between excitation and inhibition in the CNS [14]–[17].

The majority of currently used general anesthetics potentiates neurotransmission via the GABA_A_ receptor complex and/or inhibit glutamatergic signaling via the blockade of NMDA receptors [18]. Given the important role of GABAergic and glutamatergic signaling during brain maturation [19], an intriguing possibility is that exposure to general anesthetics during critical periods of development might interfere with neural circuitry assembly. We tested here this hypothesis by examining spine density and dynamics following application of anesthetics or by applying antagonists of excitatory receptors. Using in vivo and in vitro analyses, we find that these pharmacological approaches lead to a rapid regulation of spine and synapse number during critical periods of cortical development. We show that this effect (i) is produced through an enhanced rate of spine and filopodia growth and a better long-term stabilization of newly formed spines, (ii) is lasting and (iii) results in the formation of functional synapses. Altogether, these results reveal that general anesthetics-induced modulation of neural activity initiates substantial changes in synapse number and dynamics, shaping thereby cortical connectivity during critical periods of development. Importantly, these new data also raise essential questions with regard to the debate about the safety and cognitive consequences of administering anesthetics to young infants.

## Results

To examine the role of the general anesthetics on spine dynamics, we carried out both in vivo and in vitro experiments using different pharmacological tools including anesthetics which either enhance GABAergic transmission (midazolam, propofol) or interfere with excitatory NMDA dependent responses (ketamine). For in vivo analyses, spine density and morphology were analyzed using the transgenic H-line mice expressing the yellow fluorescent protein (YFP) in distinct subsets of cortical and hippocampal neurons from the second postnatal week [20]. Mice were subjected to a 5 h anesthesia at different ages and then sacrificed, fixed through perfusion and spine characteristics analyzed.

**Figure 1:**
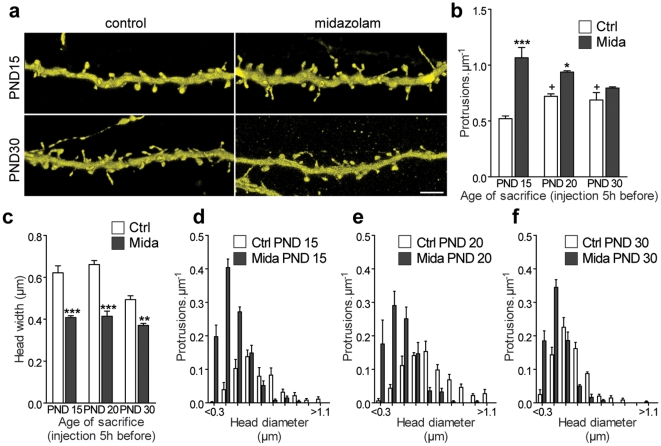
Midazolam increases protrusion density and reduces protrusions head width in the somatosensory cortex *in vivo*. (a) Representative confocal images (3D volume rendering) showing apical dendritic shafts in control and midazolam-treated animals at PND 15 and PND 30. Scale bar: 5 µm. (b) Protrusion density of pyramidal neurons of the SCC of mice sacrificed at PND 15; 20 and 30 (ctrl; +: P<0.05, Kruskal-Wallis with Dunn post tests). The increase in protrusion density is age-dependent in midazolam injected animals (Mida) compared to control groups (*n* = 4 animals per group, 4726 protrusions; two-way ANOVA with Bonferroni post tests). (c) Mean dendritic spine head diameter for midazolam-injected (Mida) compared to non-anesthetized mice (Ctrl) at PND 15, 20 and 30 (*n* = 4 animals per group, 1695 protrusions; ***: *P*<0.001, **: *P*<0.01, two-way *ANOVA* with Bonferroni post tests). (d–f) Frequency distribution histogram of spine head diameter shows that the effects observed in (b, c) are primarily due to an increase in the number of spines with small heads (each thick mark on x axis is a 0.1 µm interval).

In mice that did not undergo anesthesia, we found, consistent with previous reports [21]–[23], that there was a significant increase in protrusion density on tufted apical dendrites of layer 5 pyramidal neurons of the somatosensory cortex (SSC) between postnatal day (PND) 15 and 30 (**Fig. 1a, b**). Dendritic protrusion density increased by 38±5% from 0.52±0.02 to 0.72±0.02 protrusions µm^−1^ between PND 15 and PND 20 (Ctrl, open column: *P*<0.05), and then remained unchanged at PND 30 (0.69±0.07). At PND 15, 7.6±2% of dendritic protrusions were long and thin filopodia, and their proportion progressively decreased to 3.75±1% by PND 30 (*P*<0.001).

In marked contrast, animals sacrificed at the end of a 5 h general anesthesia made with midazolam (25 mg/kg) at PND 15 showed a dramatic, two-fold increase in protrusion density with regard to non-anesthetized mice (**Fig. 1a, b**). This density increase concerned both dendritic spines (0.48±0.01 in control vs 0.94±0.06 µm^−1^ in midazolam group) and filopodia (0.04±0.02 in control vs 0.16±0.01 µm^−1^ in midazolam group). The higher protrusion density observed in the midazolam group was associated with a significant decrease in mean spine head diameter (0.62±0.03 µm, control vs 0.41±0.01 µm, mida, *P*<0.001 **Fig. 1c**). This effect was primarily due to an important increase in the number of spines with smaller head diameters, representing, most probably, newly formed spine populations (**Fig. 1d**). Thus a 5 h-long anesthesia with midazolam at PND 15 was sufficient to profoundly increase protrusion density on tufted shafts of layer 5 pyramidal neurons.

We then tested whether anesthesia produced similar effects at different ages by exposing mice to a 5 h midazolam anesthesia at PND 20 and 30. As illustrated in **Fig. 1b**, midazolam still induced a significant, but smaller increase in protrusion density on apical tufted dendrites at PND 20 (30±2% at PND 20 vs 104±20% at PND 15; *P*<0.001). At PND 30 however, midazolam anesthesia no longer modified protrusion density (**Fig.1a, b**). Mean spine head diameters were significantly reduced following a 5 h midazolam anesthesia at PND 20, and less at PND 30 (mean decrease at PND 15: 35±1.2%; at PND 20: 37±4%; at PND 30: 10±2%; *P*<0.01, **Fig. 1c**). At all age groups, midazolam-induced decrease in average spine head diameter was primarily due to an increase in the number of spines with smaller head diameters (**Fig. 1d–f**). Together, these data suggest that the impact of anesthesia on dendritic spines is dependent on developmental age.

**Figure 2:**
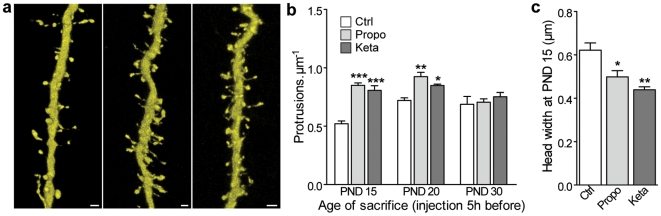
Effects of propofol and ketamine anesthesia on dendritic protrusions in the somatosensory cortex *in vivo*. (a) 3D volume rendering of confocal microscopy images of apical dendrites in control (left), propofol-treated (middle) and ketamine-treated (right) PND 15 mice. Scale bars: 1 µm. (b) Protrusion density of pyramidal neurons of the SCC of mice sacrificed at PND 15; 20 and 30 just after a 5 h-anesthesia with propofol (Propo) or ketamine (Keta) compared to control conditions (ctrl). (c) spine head width at PND 15 after 5 h-propofol (Propo) or Ketamine (Keta) anesthesia compared to control group. (b, c): *n* = 4 animals per group, 6945 spines; ***: *P*<0.001, **: *P*<0.01, *: *P*<0.05, two-way *ANOVA* with Bonferroni post tests.

We then wondered whether other anesthetics also reproduced these effects. For this, we used propofol (50 mg/kg), another GABA_A_ receptor agonist, and ketamine (30 mg/kg), an NMDA receptor antagonist. Similarly to midazolam, these two drugs also induced an age-dependent significant increase in dendritic spine density (**Fig. 2a, b**, *P*<0.05) as well as a simultaneous decrease in average dendritic spine head diameter (**Fig. 2c:** PND 15, *P*<0.05). Altogether, these results strongly suggest that several general anesthetics, affecting the excitation/inhibition balance through different mechanisms, can alter in a similar fashion the development of dendritic spines.

**Figure 3:**
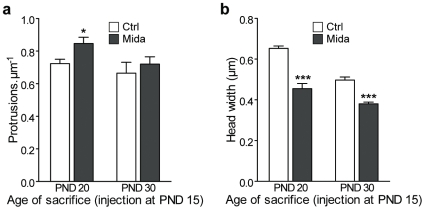
Effects of midazolam on protrusion density and on head width lasts for several days. (a) A 5 h midazolam anesthesia is performed at PND 15, protrusion density is measured at PND 20 in one group and at PND 30 in another group. (b) Protrusion head width at PND 20 and PND 30 compared to control groups. (b, c): *n* = 4 animals per group, 1571 spines; ***: *P*<0.001, *: *P*<0.05, two-way *ANOVA* with Bonferroni post tests.

To examine if the anesthetics-induced changes were lasting, mice received midazolam anesthesia at PND 15 and were then sacrificed at PND 20 and 30. At PND 20, dendritic protrusion density on apical tufts was still significantly higher in animals that received midazolam 5 days before than in aged-matched non-anesthetized controls (**Fig. 3a** **,** *P*<0.05). At PND 30 however, no such difference could be detected. Note that the increase in spine density promoted by anesthesia at PND 15 is sufficiently lasting that it now suggests a pruning effect between PND 15 and PND 30, while protrusion density in non-anesthetized animals actually increased during this period. Anesthesia-induced changes in spine head diameter, observed following a 5 h midazolam anesthesia at PND 15, was still present at PND 20 (*P*<0.001) and, although to a lesser extent, also at PND 30 (mean decrease compared to age-matched controls at PND 20: 30±4.5%; at PND 30: 23±2.1%; **Fig. 3b**). The changes induced by a general anesthesia in young animals may thus last for several days.

**Figure 4:**
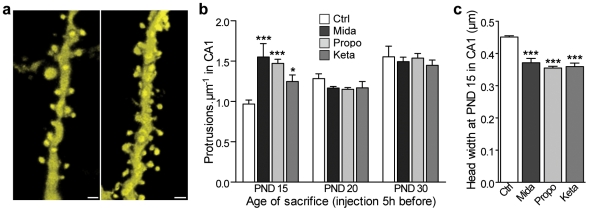
Effects of midazolam, propofol and ketamine on dendritic protrusions in the hippocampus *in vivo*. (a) 3D volume rendering of confocal microscopy images of apical dendrites of CA1 hippocampal neurons of PND 15 mice in control (left) and midazolam-anesthetized (right) conditions. Scale bars: 1 µm. (b) Protrusion density of pyramidal neurons of mice sacrificed at PND 15; 20 and 30 just after a 5 h-anesthesia with midazolam (Mida) propofol (Propo) or ketamine (Keta) compared to control conditions (ctrl). (c) protrusion head width in PND 15 mice CA1 neurons after 5 h-midazolam (Mida), propofol (Propo) or Ketamine (Keta) anesthesia compared to control group. (b, c): *n* = 4 animals per group, 1291 spines; ***: *P*<0.001, *: *P*<0.05, two-way *ANOVA* with Bonferroni post tests.

We then wondered whether these changes were specific to the SSC, and therefore evaluated the effects of these three anesthetics on the development of dendritic protrusions in another cortical region, area CA1 of the hippocampus, where cells also express YFP. At PND 15, we found that all anesthetics induced within 5 h a significant increase in protrusion density on apical dendrites of CA1 pyramidal neurons (**Fig. 4a, b** **,** *P*<0.05). The effect was associated with a concomitant increase in the percentage of spines with smaller head diameter (**Fig. 4c** **,** *P*<0.001). Similarly to observations made in the SSC, the effects of the three anesthetics on CA1 dendritic spines were dependent on the age of the animals (**Fig. 4b**), thus reproducing the changes observed in the SSC.

**Figure 5:**
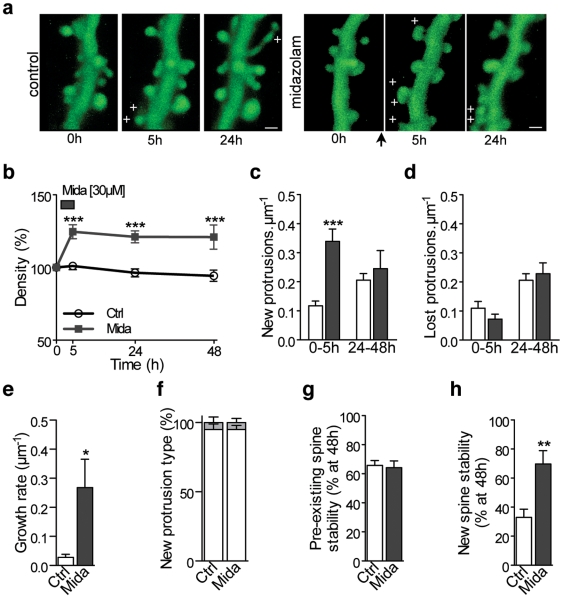
Spine dynamics underlying midazolam-induced increase in spine density. (a) Maximum intensity projections with 3D volume rendering of confocal images of a CA1 dendrite observed repetitively at 0 h; 5 h and 24 h showing the protrusion turnover in control conditions (left panel) and after midazolam (right panel). +: newly formed protrusions. Scale bar: 1 µm. Hippocampal slices were incubated with midazolam (30 µM) during 5 h just after the first observation (arrow). (b) Protrusion density (expressed as percent of initial values) observed at 0 h, 5 h, 24 h and 48 h in control condition (empty circle) and after midazolam treatment (dark squares). (c) New protrusions per µm detected between 0 to 5 h, or between 24 to 48 h in control conditions (white bars) and after midazolam treatment (dark bars). (d) Same as (c), but for lost protrusions. (e) Number of protrusions per µm that appeared after 1 hour midazolam incubation compared to control conditions. (f) Type of new protrusions for control (ctrl) and midazolam (mida). White bars: spines; gray bars: filopodia. (g) Fraction of spines present at the initial observation (pre-existing spines) still present at 48 h. (h) Fraction of new spines formed between 0 h and 5 h and still present at 48 h. Note that in (g) and (h), only spines were considered: filopodia were excluded because of their very short-term survival. (b–d; f–h): *n* = 12; 13 cultures for control and midazolam conditions, respectively, 1726 protrusions analyzed. (e): *n* = 4 cultures for each conditions, 293 protrusions analyzed. (b): 2-way *ANOVA* with Bonferroni post tests, (c–h): unpaired *t* tests.

To further investigate the dynamics and mechanisms underlying these effects, and since in vivo imaging is precluded by the need of anesthesia, we performed repetitive confocal imaging of eGFP transfected CA1 pyramidal neurons in 2–3 weeks old hippocampal slice cultures, a model also showing a developmental regulation of spine turnover [24]. We found that a 5-hour-long bath application of midazolam (30 µM) rapidly induced a significant increase in protrusion density that still persisted 48 hours after application (**Figure 5a, b** **,** *P*<0.001). To understand the dynamic changes underlying this effect we measured the proportion of newly formed and lost protrusions detected on identified dendritic segments between observations made at 0, 5, 24 and 48 hours. We found that midazolam triggered a striking increase in protrusion formation (**Fig. 5c** **, 0–5 h,** P<0.001), whereas the loss of protrusions remained unchanged (**Fig. 5d**, **0–5 h,** *P*>0.05). This effect was however transient and limited to the presence of midazolam, since 24 h after removal of midazolam, the rate of new spine formation and loss was again similar to those found in non-treated controls (**Fig. 5b, c:** **24–48 h,** *P*>0.05). We also determined the number of newly formed spines per µm after one hour-treatment with midazolam. This growth rate was more than 9 times higher compared to control conditions (26.8±9.7 vs 2.8±1.1 new spines per µm per hour, respectively, **Fig. 5e**). Interestingly, the proportion of filopodia among new protrusions was not changed and remained low (**Fig. 5f** **,** *P*>0.05). We then also analyzed how midazolam treatment affected spine stability by measuring the proportion of spines persisting over a period of 48 h. Pre-existing spines, present before the application of midazolam, were not affected in their stability by midazolam treatment, indicating that the drug probably did not modify pre-established circuits (**Fig. 5g** **,** *P*>0.05). However, spines formed in the presence of midazolam had a two times higher probability to remain stable than newly formed spines appearing during the same period in control untreated cultures (**Fig. 5h** **,** *P*<0.01). These data indicate that midazolam-induced increase in protrusions density is primarily due to a rapid increase in spinogenesis followed by an enhanced stabilization of newly formed protrusions. These combined, but transient effects probably account for the fact that the midazolam-induced increase in spine density lasted for several days.

**Figure 6:**
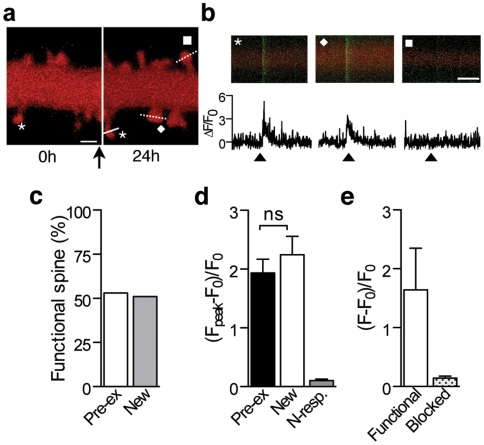
New spines formed following midazolam treatment are functional. (a) 3D volume rendering of images of an mRFP transfected and Fluo-4 AM loaded cell showing newly formed spines detected 24 h after midazolam application (arrow). The dashed lines and the plain line indicate where line scan analysis was performed at 24 h for the 2 new spines (square and diamond) and a pre-existing spine (star), respectively. (b) Top: line scans of the spines with the corresponding symbols in (a), the red channel showing mRFP fluorescence, and the green channel showing Fluo-4 AM fluorescence. Bottom: Fluo-4 AM corresponding signals expressed as ΔF/F_0_, illustrating the intracellular calcium response obtained upon electrical stimulation of Schaffer collaterals (arrowhead). (c) Percentage of spines that responded to stimulation by an elevation of intracellular free calcium concentration for new (grey) and pre-existing spines (white). (d) Mean amplitude of calcium responses (ΔF/F_0_ measured at the peak of the response triggered by electrical stimulation of Schaffer collaterals) for spines present at the beginning of the experiment (pre-existing spines, *n* = 15 spines in 2 neurons) compared to spines that appeared during of after midazolam treatment (new spines, *n* = 43 in 4 neurons). *P* = 0.65, unpaired *t* test. N-resp.: calcium signal in non responding spines (*n* = 23 in 4 neurons). (e) Calcium signal of newly formed functional spines before (Functional) and after (Blocked) a half an hour bath application of AP5 (50 µM) and NBQX (20 µM) (*n* = 8 new spines in 2 cells). Compare to the mean signal recorded for non responding spines in (d). F corresponds to the mean amplitude of the Fluo-4 fluorescence measured during the 20 ms consecutive to the stimulation pulse. Scale bars: (a): 1 µm. (b): 2 s.

We next tested if new spines formed following midazolam application could be functional. After bolus loading of cells with the calcium indicator Fluo-4 AM, functionality of the new spines detected 24 h after midazolam application was tested by stimulation of Schaffer collaterals and recordings of calcium signals in individual spines (**See Methods**). Out of 43 new spines tested (4 cells), 22 responded to stimulation by an increase in intracellular calcium (**Fig. 6a, b**). Spines that did not show calcium responses were thus either silent, non-functional or more simply, the presynaptic axon could not be stimulated through the depolarizing current pulses. Under control conditions, the same proportion of spines also failed to show calcium responses (**Fig. 6a–c**), indicating that newly formed spines have the same probability to be functional as pre-existing spines. Furthermore, the responses of newly formed spines were comparable in amplitude with responses of neighboring pre-existing spines (**Fig. 6d**), and were abolished by blocking fast glutamatergic transmission with AP5 and NBQX (**Fig. 6e**). Therefore, a significant fraction of spines formed after midazolam treatment are functional and integrated in the pre-existing network within 24 h, thus probably accounting for the long-lasting effect on spine density of anesthetics.

**Figure 7:**
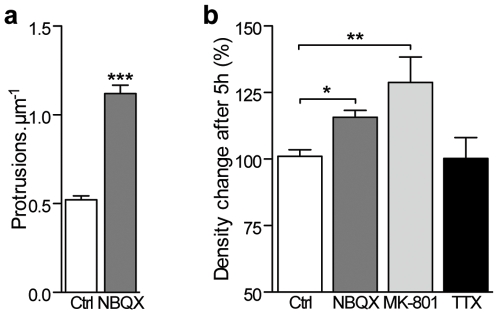
Selective blockade of ionotropic glutamatergic receptors induce an increase in protrusion density. (a) *In vivo*, intraperitoneal injections of NBQX (30 mg/kg) at PND 15 induced a highly significant increase in protrusion density within 5 hours (*n* = 617 spines from 3 animals in the control group and 472 spines from 6 animals in the NBQX group). (b) Effects of a 5 h bath incubation of NBQX (20 µM, *n* = 192 spines in 4 cultures), MK-801 (100 µM, *n* = 409 protrusions in 8 cultures), or TTX (1 µM, *n* = 249 protrusions in 5 cultures) on protrusion density compared to control conditions in hippocampal slice cultures (*n* = 537 protrusions in 10 cultures). (a, b): ***: *P*<0.001, **: *P*<0.01, *: *P*<0.05, unpaired *t* test.

Finally, we evaluated whether exposure to specific inhibitors of ionotropic glutamate receptors also increased protrusion density. As seen on **Figure 7a**, in vivo intraperitoneal administration of the AMPA/kainate antagonist NBQX (30 mg/kg) to PND 15 mice rapidly and significantly increased the number of protrusions in the SSC (*P*<0.001). In line, in hippocampal slice culture, a 5 h incubation with NBQX (20 µM) also induced an increase in protrusion density (**Fig. 7b**, *P*<0.05). In addition, blockade of NMDA receptor with MK801 (100 µM) also increased protrusion density within 5 h (**Fig. 7b**, *P*<0.01) whereas simultaneous blockade of both excitatory and inhibitory activity by TTX (1 µM) did not reproduce the effect (**Fig. 7b**). Taken together, these results further suggest the importance of the balance between excitation and inhibition rather than activity per se in the regulation of spinogenesis.

## Discussion

The present study provides evidence that exposure to general anesthetics rapidly induces dendritic spine growth and the formation of functional synapses during critical periods of early postnatal life in the rodent brain. This is to our knowledge the first demonstration that a few hours of anesthesia, can almost double the spine density on cortical neurons and thus the complexity of cortical synaptic networks. The magnitude of the effect, its persistence over several days and its restriction to critical periods of development certainly raises important questions as to the mechanisms involved, but also regarding the possible functional consequences of such a massive synaptic reorganization. The fact that both anesthetics that enhance GABA_A_ receptor-mediated inhibition and those blocking NMDA receptor-mediated excitation promoted spine growth suggests that the balance between excitation and inhibition was the major determinant of this synaptic growth process. This could be in line with previous results indicating changes in spine and synapse properties or number upon prolonged interference with excitatory or inhibitory receptors, [25]–[30]. However neither the context, nor the dynamics of these previously reported changes makes them really comparable to our study, and clearly more experiments will be required to identify the signaling mechanisms implicated in these regulations.

The increase in spine and synapse density reported here following reduction of excitation in the tissue points to similarities with the homeostatic scaling effect described for excitatory receptors where pharmacological blockade of excitation promotes expression of additional receptors at all synapses [31], [32]. In both cases the regulations involved tend to maintain neuronal activity, either by increasing sensitivity to glutamate at existing synapses, or as shown here, by increasing synapse number. There are however also different specificities between these mechanisms. The synaptic changes reported here are surprisingly fast and predominantly observed during a critical period of development, unlike the receptor scaling effect and unlike the changes in spine density or properties reported following long-term, chronic blockade or genetic manipulation of NMDA or AMPA receptors [25], [27], [29]. Additionally, unlike receptor scaling [32], spine growth triggered by interference with the excitation/inhibition balance is achieved by blockade of both AMPA and NMDA receptors, indicating that it is very sensitive to NMDA dependent mechanisms. The fast regulation of synapse number reported here could thus maintain the level of excitatory activity required for ensuring plasticity-mediated mechanisms. This could be particularly important at times where synapse turnover is high and selection of correct inputs is a central issue. This could also provide some clue regarding the physiopathological mechanisms possibly relating modulation or shifts of the excitatory/inhibitory balance to neuropsychiatric disorders such as proposed in Down or Rett syndromes as well as in autism spectrum disorders [14]–[17].

There are two additional important aspects related to this homeostatic mechanism. First, the magnitude and rapidity of the effect produced probably account for some of the discrepancies reported in the literature concerning the development of spines during the first weeks of life. Recent studies using in vivo 2-photon imaging have reported mechanisms of spine pruning between the second, third and fourth week of life in mice [33]. This is however at variance with previous anatomical data obtained through classical staining and brain fixation methods that reported a continuous increase in spine density in many cortical regions during the same periods [21]–[23]. Interestingly, in vivo imaging studies have been carried out in mice that had undergone a long anesthesia for the preparation of 2-photon imaging approach. This manipulation probably boosted spine density in these young mice and, as shown by our data, analyses in animals anesthetized around PND 15 then suggest a subsequent pruning of spines over the next 2 weeks, while analyses of mice that did not undergo anesthesia conversely indicate a progressive increase in spine density. Accordingly, our data indicate that anesthesia in young animals may induce important structural modifications that might be source of misinterpretations regarding analyses of spine number or spine morphology.

The second important aspect of this work relates to the effects that anesthetics might produce in human clinical use when applied during critical periods of development in infants. Our work shows that all anesthetics tested, which all interfere with the excitation/inhibition balance, promote a rapid increase in spine synapse density, but also affect spine morphology. These two effects were lasting for several days in young mice, and certainly contributed to modify cortical networks since many new spines turn out to be functional synapses. Although the behavioral significance of these changes remains to be determined, they might raise concern about the millions of human infants that receive general anesthesia during this developmental period every year worldwide. Indeed, an increasing number of clinical reports suggest the possibility of adverse long-term neurocognitive outcome in the population of young infants undergoing anesthesia/surgery [34]–[36].

Altogether, this study demonstrates that exposure to general anesthetics during critical periods of development increases dendritic spine number and suggests a mechanism for the rapid modulation of synaptogenesis via the modulation of the excitation/inhibition balance by these drugs. This new mechanism is likely to play a critical role in the regulation of the formation of neural circuits and may help understand dysfunctions related to conditions under which alterations of the excitation/inhibition balance may occur.

## Materials and Methods

### Ethics statements

All procedures were done in accordance with the animal care and use guidelines of the Geneva Cantonal Veterinary Office and the University of Geneva.

### Experimental animals and anesthesia procedure

Mice expressing the yellow fluorescent protein (YFP) in layer 5B cortical neurons (H-line) [20] [37] were purchased from the Jackson laboratory (Bar Harbor, Maine, USA). Animals were group-housed and bred in the animal facilities of the University of Geneva Medical School under light (12 h light/dark cycle) and temperature (22±2°C) controlled conditions. Food and water were available *ad libitum*. Every effort was made to minimize the number of animals used and their suffering.

General anesthesia was induced by intraperitoneal injection of midazolam (25 mg/kg), propofol (50 mg/kg) or ketamine (30 mg/kg). At these concentrations, these drugs induced deep sedation (absence of the righting reflex >10 s) for approximately 90 minutes. Subsequent injections of midazolam, propofol and ketamine at concentrations of 15 mg/kg, 25 mg/kg and 15 mg/kg respectively, allowed maintaining anesthesia for an additional 90–120 minutes. Thus, to provide a 5-hour-long anesthesia, animals received a total of 3 intraperitoneal injections of anesthetics at 90 minutes intervals. To ensure that this abovementioned anesthesia procedure did not induce significant metabolic and/or respiratory compromise, we performed arterial blood sampling from the ascending aorta in three PND 16 animals of each anesthesia protocol at the end of the 5-hour-long anesthesia procedure. This analysis revealed that physiological parameters, such as pO2, pCO2, lactate and blood sugar concentrations remained within physiological range. Control sham-treated animals received 3 intraperitoneal injections of physiological saline at 90 minutes intervals and have undergone the same handling and maternal separation as anesthetized animals. In a set of preliminary experiments we determined that this “sham treatment paradigm” does not induce any changes in dendritic spine density or morphology in the somatosensory cortex compared with control non-treated animals sacrificed immediately following maternal separation. To avoid hypothermia, animals were kept during the whole procedure on a homeothermic blanket system in order to maintain body temperature at 37°C (Harvard Apparatus, Holliston, Massachusetts, United States).

### Histological processing and immunohistochemistry

Depending on experimental protocols, animals were sacrificed either immediately following the 5-hour-long anesthesia period or at later time points, as indicated, using a lethal dose of pentobarbital (100 mg/kg) followed by intracardial perfusion of a cold (4°C) 0.9% saline and, subsequently, by a 4% paraformaldehyde solution. Brains were then extracted from the skull and post-fixed overnight in a 4% paraformaldehyde solution at 4°C. Following fixation, brains were washed 3×1 hour in a 0.1 M phosphate-based saline (PBS; pH 7.2) solution and then cryoprotected with sucrose (30%). 20 µm-thick sections were cut using a cryostat (Leica, Wetzlar, Germany) and plated on gelatin-coated slides. To strengthen signal intensity of YFP-positive dendritic shafts, sections were incubated overnight at 4°C with a polyclonal rabbit anti-GFP antibody (1∶1000; Molecular Probes, Invitrogen, Carlsbad, California, United States) in PBS/0.5% bovine serum albumine (BSA)/0.3% Triton X-100. Sections were then rapidly washed 3 times in PBS and incubated for 90 minutes with an Alexa-conjugated secondary antibody (Molecular Probes, Carlsbad, California, United States) diluted in PBS/0.5% bovine serum albumine (BSA).

### Organotypic hippocampal slice cultures

Transverse hippocampal organotypic slice cultures (400 µm thick) from 6–7 day-old rats were prepared as described [38], using a protocol approved by the Geneva Veterinarian Office (authorization 31.1.1007/3129/0) and maintained for 11–18 days in a CO_2_ incubator at 33°C. Transfection was done with a pc-DNA3.1-EGFP plasmid using a biolistic method (Helios Gene Gun, Bio-Rad) 3 days before the first observation. Fluorescence usually started to be expressed after 24–48 h and then remained stable for at least 15 days. The use of rat slice cultures rather than YFP mouse tissue was justified by technological reasons related to the preparation and transfection of slice cultures allowing to visualize single transfected cells, the existence of numerous previous data regarding spine dynamics in this preparation and the interest of being able to confirm in a different animal model the main observations.

### Confocal imaging

#### Fixed tissue

A Zeiss LSM 510 meta confocal microscope (Carl-Zeiss, Göttingen, Germany) equipped with a 63×oil-immersion objective was used to obtain images from apical dendritic tufts of layer 5 pyramidal neurons (1^st^ 100 µm from the pial surface) in the somatosensory cortex and from apical dendrites of CA1 pyramidal neurons (focusing on secondary and tertiary dendrites located between 100 and 300 µm from the soma) in the hippocampus. A numeric zoom of 3.2 was used and 0.5-µm-thick sections were obtained using an appropriate wavelength (488 nm) for detecting YFP. Stack images of apical dendritic shafts with a length between 25 and 50 µm were randomly taken from 4 animals per experimental group (10 stacks/animal).

#### Hippocampal slice cultures

Short imaging sessions (10–15 min) of transfected slices were carried out with an Olympus Fluoview 300 system coupled to a single (Olympus) and a 2-photon laser (Chameleon; Coherent) as described [24]. Laser intensity in all these experiments was kept at the minimum and acquisition conditions maintained mostly unchanged over the different days of observation. Control experiments showed that transfection and repetitive confocal imaging of slice cultures did not alter cell viability over periods of weeks. We focused on dendritic segments of about 40 µm in length and located between 100 and 350 µm from the soma on secondary dendrites using a 40×objective and a 10×additional zoom (final resolution: 25 pixels per micron; steps between scans: 0.4 µm). For calcium imaging of spine activity, new spines were identified 24 h after midazolam application in mRFP transfected cells. Cells were then loaded with the cell permeable calcium indicator Fluo-4 AM (F-14201, Invitrogen). For this, 50 µg of Fluo-4 AM was dissolved in 10 µl Pluronic (F-127, Invitrogen) and then diluted in 90 µl of standard pipette solution (150 mM NaCl, 2.5 mM KCl, 10 mM Hepes) for a final dye concentration of 500 µM. A standard patch pipette was then filled with 10 µl of dye solution and placed at a distance of about 10 µm from the soma. Dye was ejected by short pulses of pressured air at a frequency of 3 per minute during half an hour. Calcium transients in identified new spines and neighboring pre-existing spines were then recorded using line scans through the spine heads obtained during application of stimulation pulses to Schaffer collaterals. Stimulating electrode was always placed at minimum at 500 µm from the dendritic tree of the Fluo-4 AM loaded cell in order to preclude direct electrical stimulation of the cell. In all experiments carried out, we also verified that spines activated by stimulation were always surrounded by other silent, non-activated spines in order to exclude global activation effects. Spines located too close to each other were excluded from analysis. Confocal aperture was set to the minimum during line scans, and matching with the mRFP fluorescence in the red channel was systematically checked. For each spine tested, 2 to 7 linescans of 8 seconds of duration were performed, with electrical stimulation pulse occurring automatically at 3 seconds. Calcium responses were measured as ΔF/F_0_, where F_0_ is the mean fluorescence measured during the baseline.

### Data analysis

In this study we refer to protrusions, whenever analyses were carried out by considering filopodia and spines. Filopodia were defined as protrusions devoid of enlargement at the tip (overall they represented less than 5% of total protrusion number), while we classified as spines all protrusions exhibiting an enlargement at the tip. All turnover and stability analyses were carried out by scrolling across single z-stacks of raw images using a plug-in specifically developed for OsiriX software (http://www.osirix-viewer.com) except for density analysis on fixed tissue, which was performed on the LSM image viewer software (Carl-Zeiss, Göttingen, Germany). The measures of density, turnover and stability were carried out by analyzing all protrusions, i.e. filopodia and spines. We counted protrusions located behind each other on z-stacks whenever distinction was possible. For turnover analysis, we counted as new protrusions all new structures (spines or filopodia) appearing between two observations (5 h or 24 h) and characterized by a length of >0.4 µm. All filopodia were counted as separate protrusions. For disappearances, we counted all protrusions (spines and filopodia) that could no longer be identified on the next observation. Dubious situations due to possible changes in protrusion shape, size or orientation were discarded, but overall accounted for only a small number of cases (less than 1%). To further ensure reliability of analyses, all measurements of spine turnover and stability were carried out blind by two experimenters. Comparisons of the analyses made in this way showed variations in the results that were less than 3%. Furthermore, we used high numbers of n for both cells and spines and labeled all new or lost protrusions directly on the raw data to allow multiple checks.

Due to the lack of survival of filopodia on several days, stability analyses only included pre-existing spines, i.e. spines present at the beginning of the experiment. Note that for illustration purposes, images presented in figures are maximum intensity projections of z stacks with volume rendering, further treated with a Gaussian blur filter.

### Statistics

All statistics are given with the standard error of the mean (s.e.m.). Normality was tested for each distribution (D'Agostino and Pearson test), and α was set to 5% for all tests.
